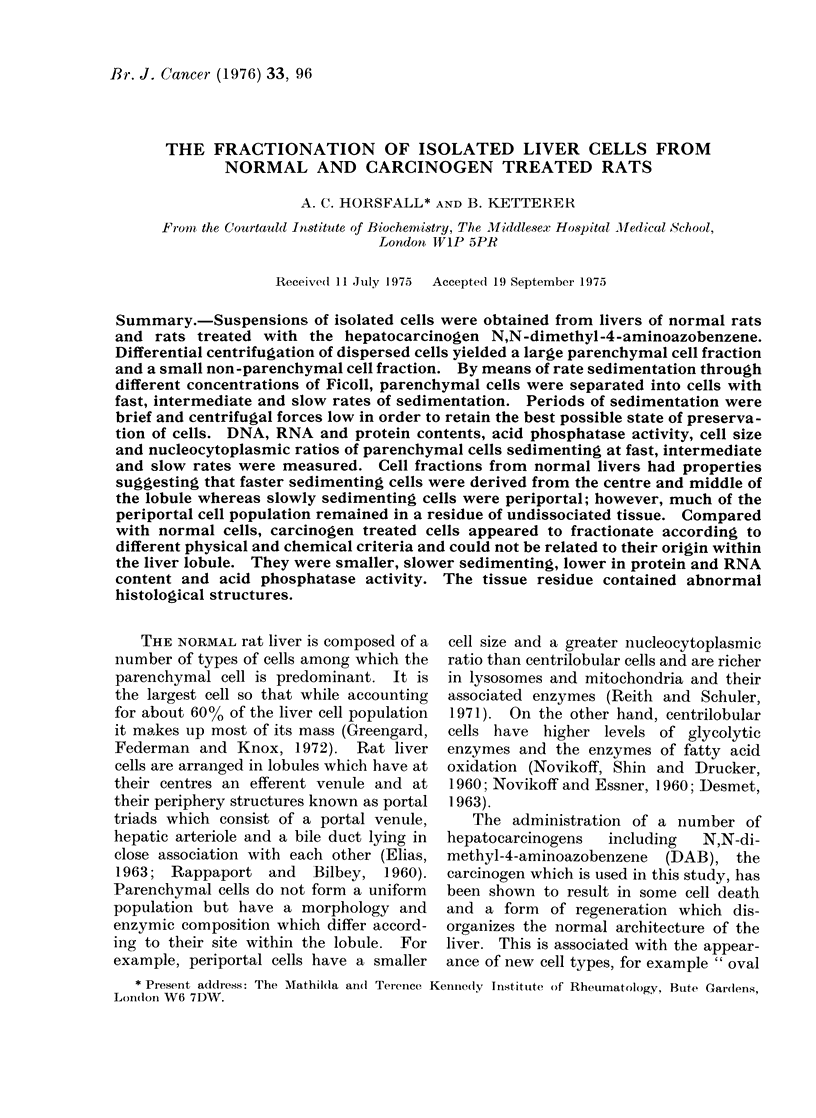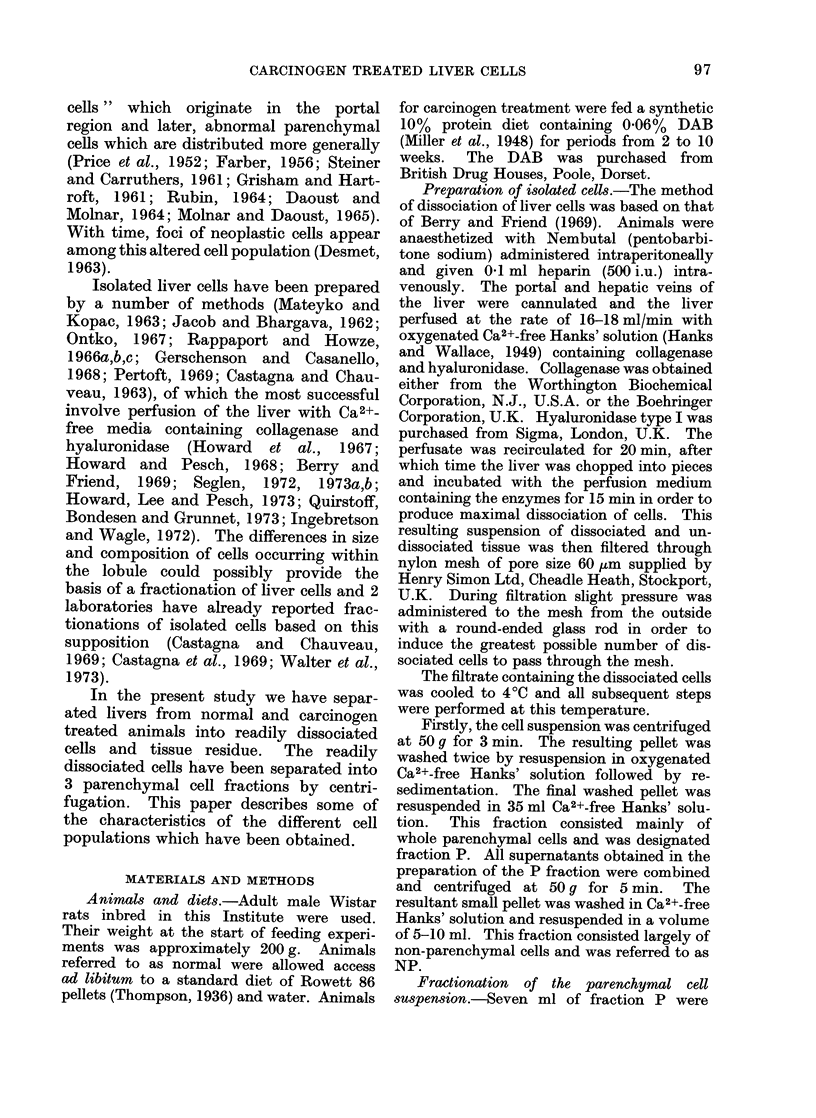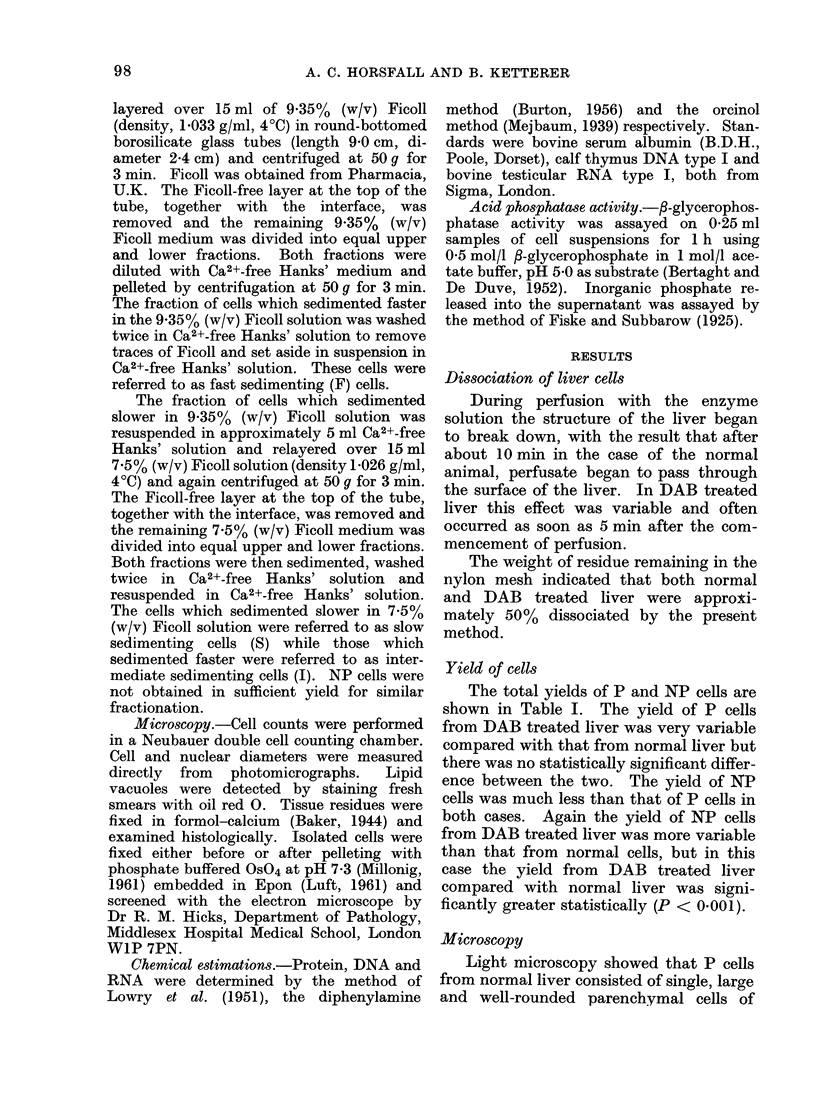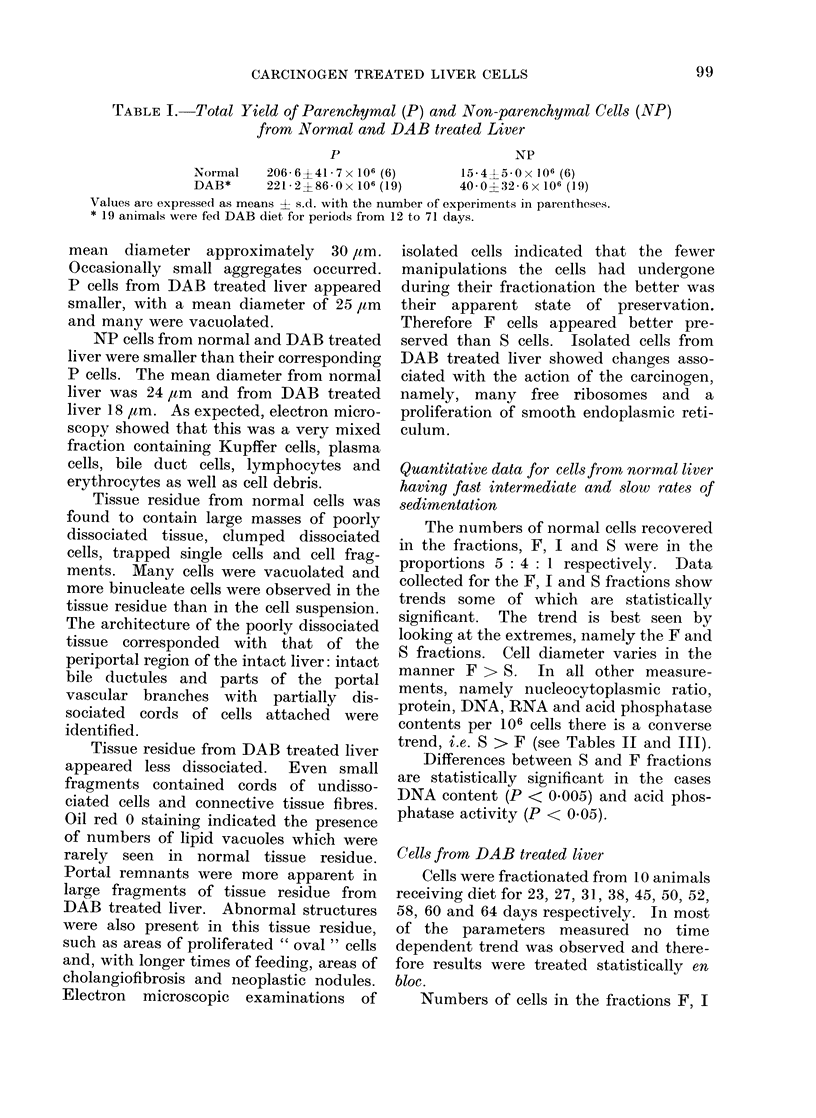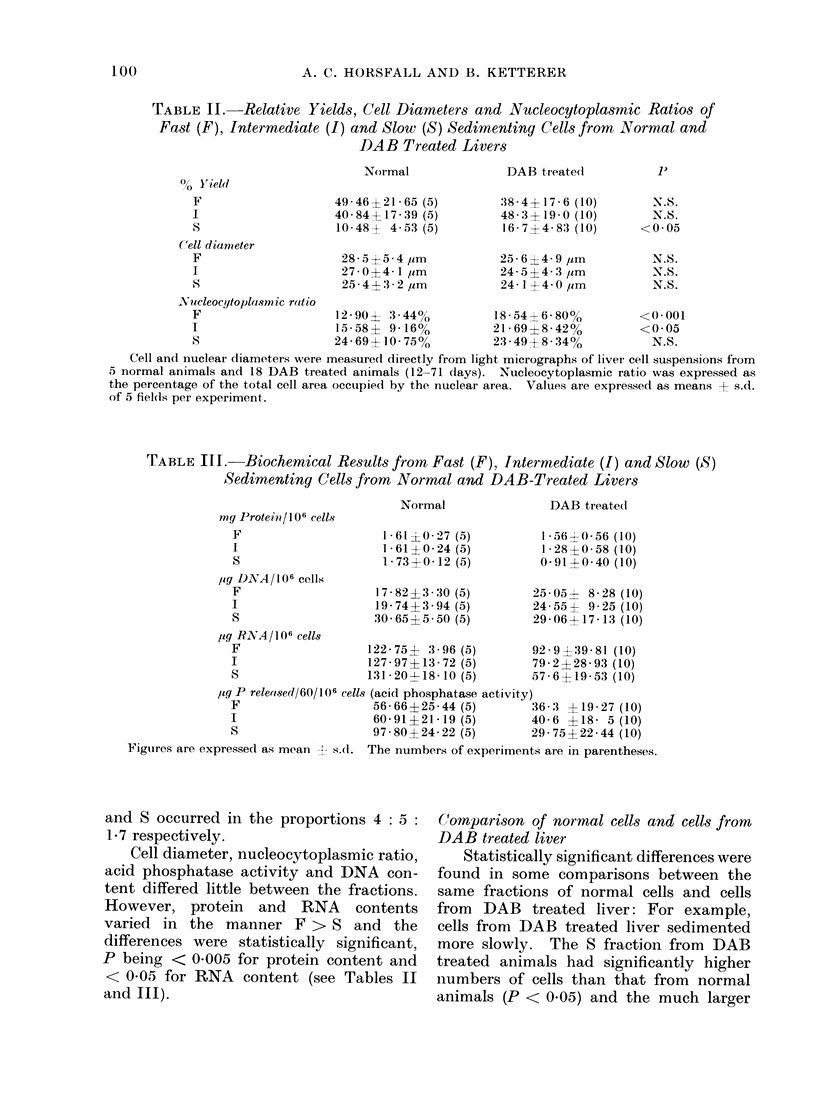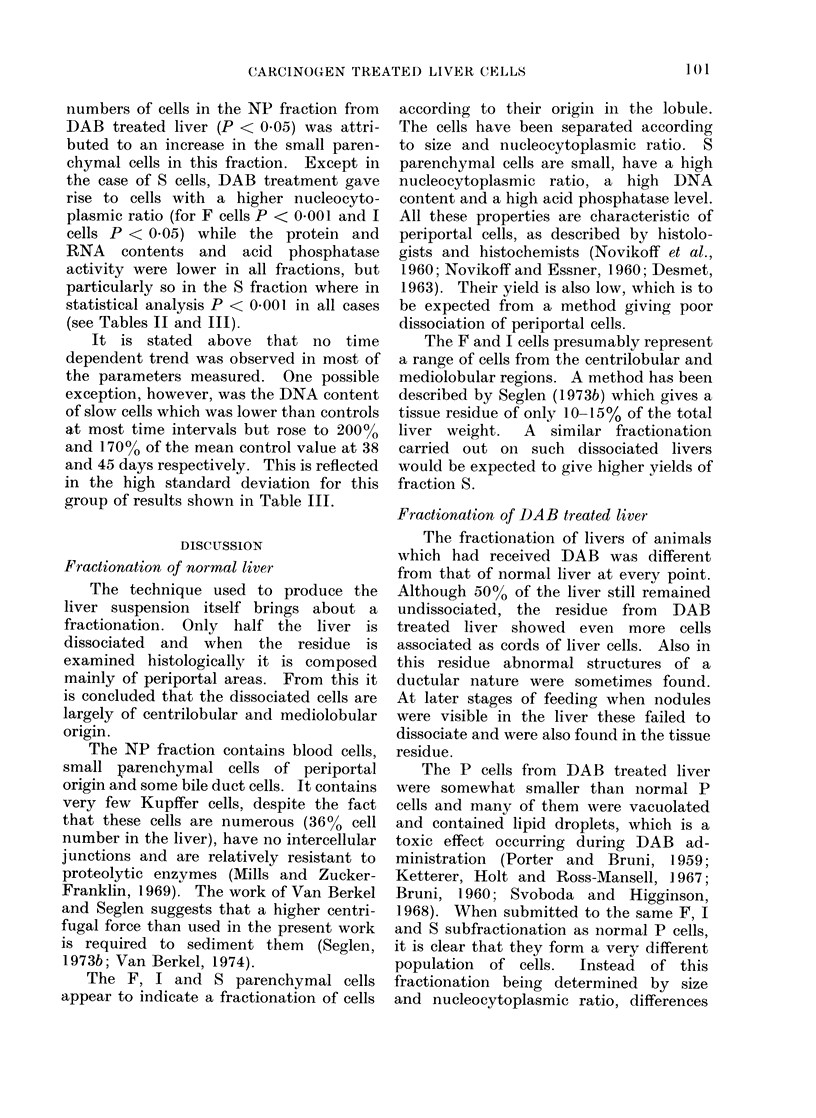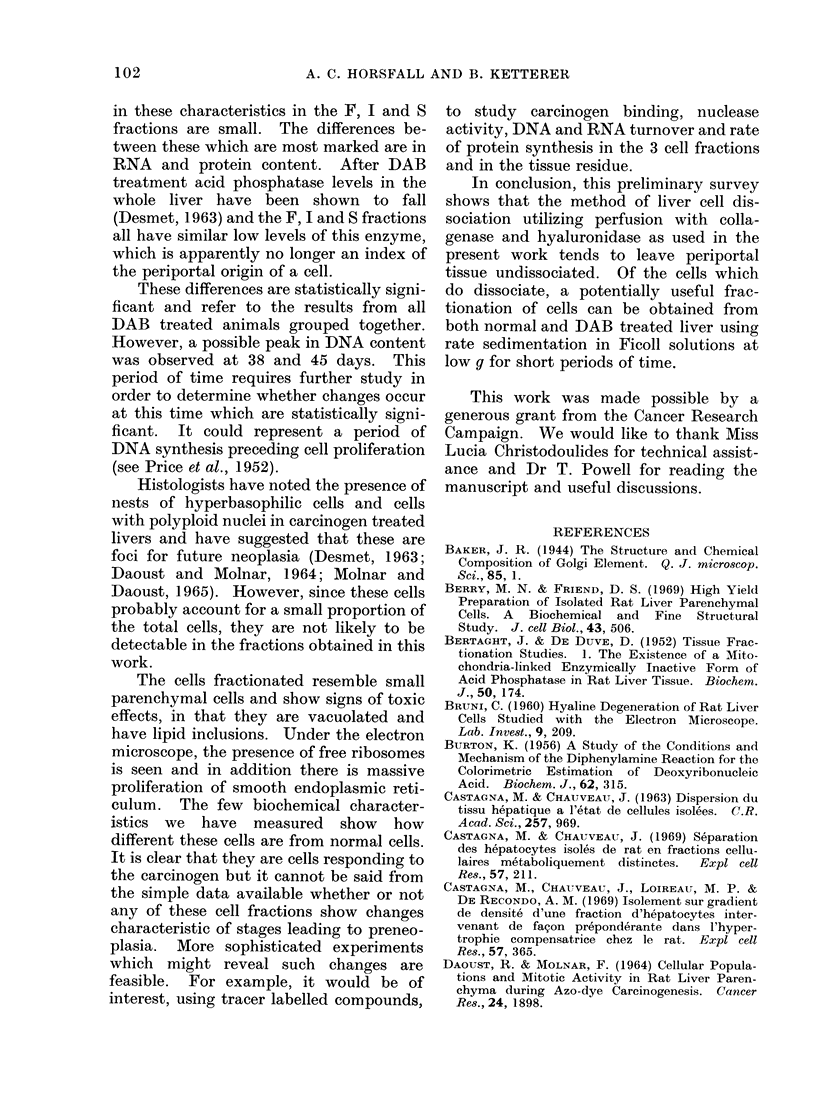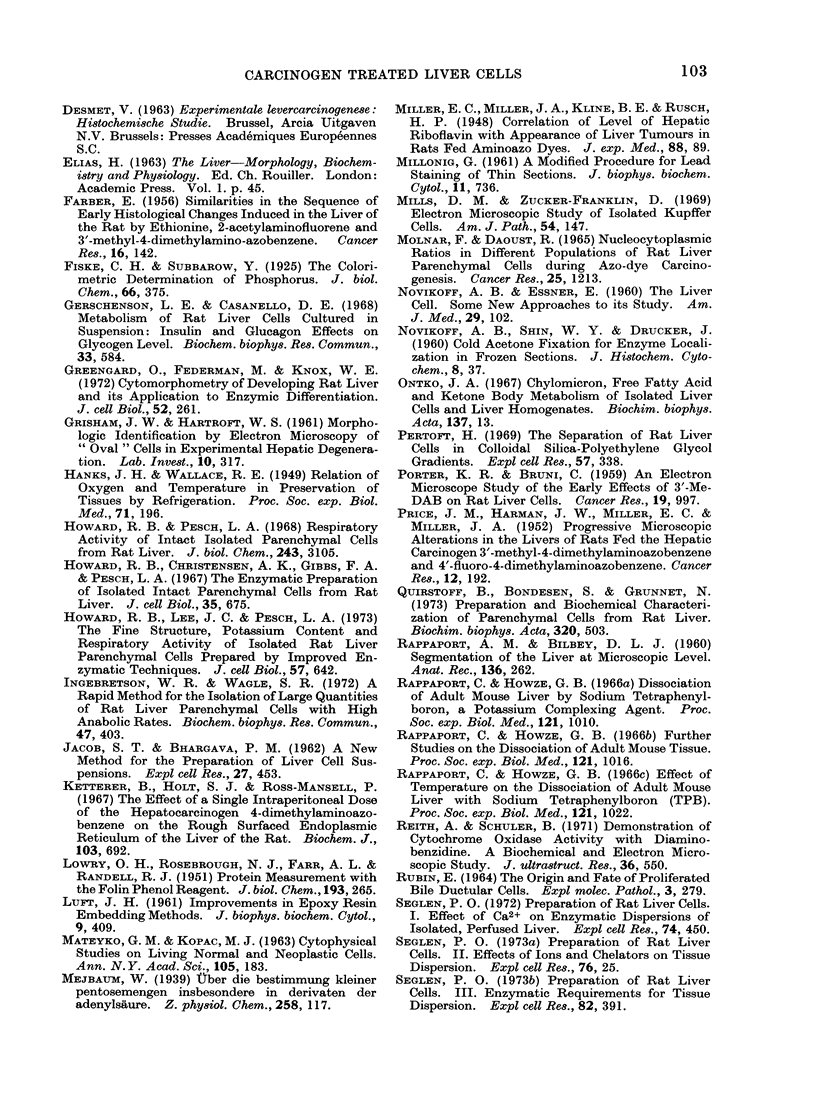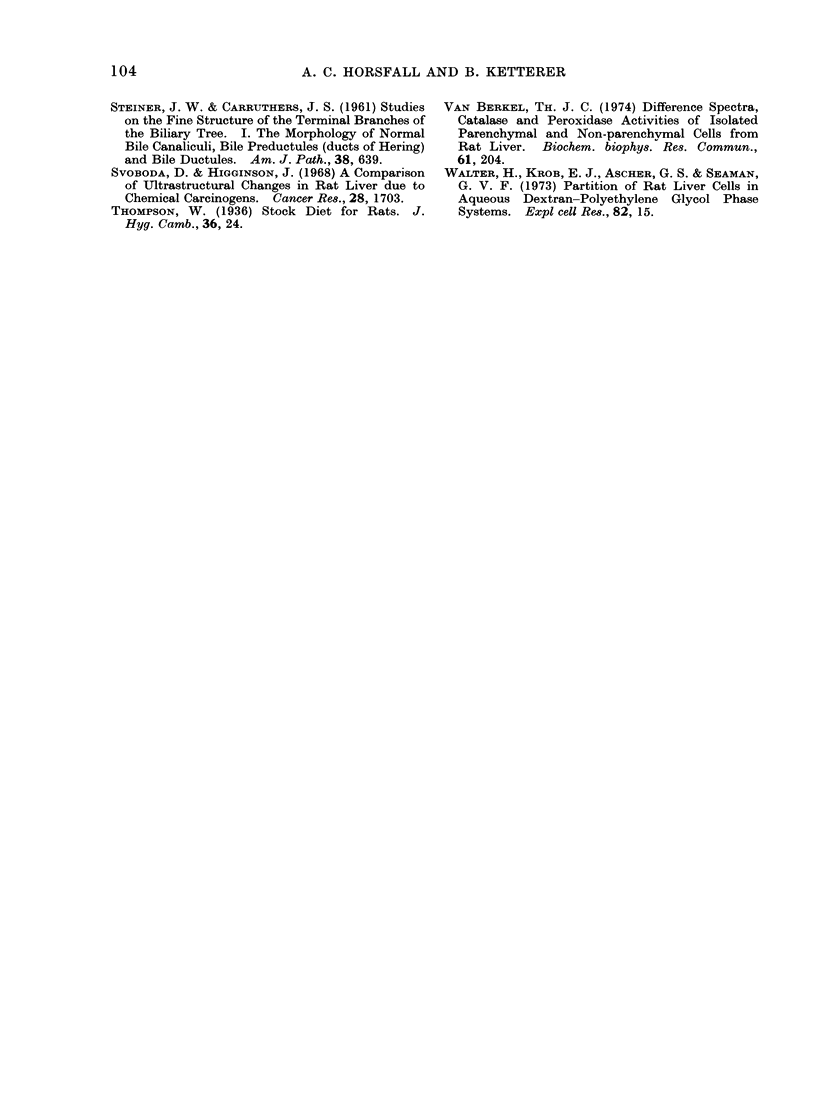# The fractionation of isolated liver cells from normal and carcinogen treated rats.

**DOI:** 10.1038/bjc.1976.10

**Published:** 1976-01

**Authors:** A. C. Horsfall, B. Ketterer

## Abstract

Suspensions of isolated cells were obtained from livers of normal rats and rats treated with the hepatocarcinogen N,N-dimethyl-4-aminoazobenzene. Differential centrifugation of dispersed cells yielded a large parenchymal cell fraction and a small non-parencymal cell fraction. By means of rate sedimentation through different concnetrations of Ficoll, parenchymal cells were separated into cells with fast, intermediate and slow rates of sedimentation. Periods of sedimentation were brief and centrifugal forces low in order to retain the best possible state of preservation of cells. DNA, RNA and protein contents, acid phosphatase activity, cell size and nucleocytoplasmic ratios of parenchymal cells sedimenting at fast, intermediate and slow rates were measured. Cell fractions from normal livers had properties suggesting that faster sedimenting cells were derived from the centre and middle of the lobule whereas slowly sedimenting cells were periportal; however, much of the periportal cell population remained in a residue of undissociated tissue. Compared with normal cells, carcinogen treated cells appeared to fractionate according to different physical and chemical criteria and could not be related to their origin within the liver lobule. They were smaller, slower sedimenting, lower in protein and RNA content and acid phosphatase activity. The tissue residue contained abnromal histological structures.


*Br. J. Cancer* (1976) **33**, 96

### THE FRACTIONATION OF ISOLATED LIVER CELLS FROM NORMAL AND CARCINOGEN TREATED RATS

A. C. HORSFALL* AND B. KETTERER

*From the Courtauld Institute of Biochemistry, The Middlesex Hospital Medical School, London W1P 5PR*

Received 11 July 1975 Accepted 19 September 1975

**Summary.—Suspensions of isolated cells were obtained from livers of normal rats and rats treated with the hepatocarcinogen N,N-dimethyl-4-aminoazobenzene. Differential centrifugation of dispersed cells yielded a large parenchymal cell fraction and a small non-parenchymal cell fraction. By means of rate sedimentation through different concentrations of Ficoll, parenchymal cells were separated into cells with fast, intermediate and slow rates of sedimentation. Periods of sedimentation were brief and centrifugal forces low in order to retain the best possible state of preservation of cells. DNA, RNA and protein contents, acid phosphatase activity, cell size and nucleocytoplasmic ratios of parenchymal cells sedimenting at fast, intermediate and slow rates were measured. Cell fractions from normal livers had properties suggesting that faster sedimenting cells were derived from the centre and middle of the lobule whereas slowly sedimenting cells were periportal; however, much of the periportal cell population remained in a residue of undissociated tissue. Compared with normal cells, carcinogen treated cells appeared to fractionate according to different physical and chemical criteria and could not be related to their origin within the liver lobule. They were smaller, slower sedimenting, lower in protein and RNA content and acid phosphatase activity. The tissue residue contained abnormal histological structures.**

THE NORMAL rat liver is composed of a number of types of cells among which the parenchymal cell is predominant. It is the largest cell so that while accounting for about 60% of the liver cell population it makes up most of its mass (Greengard, Federman and Knox, 1972). Rat liver cells are arranged in lobules which have at their centres an efferent venule and at their periphery structures known as portal triads which consist of a portal venule, hepatic arteriole and a bile duct lying in close association with each other (Elias, 1963; Rappaport and Bilbey, 1960). Parenchymal cells do not form a uniform population but have a morphology and enzymic composition which differ according to their site within the lobule. For example, periportal cells have a smaller cell size and a greater nucleocytoplasmic ratio than centrilobular cells and are richer in lysosomes and mitochondria and their associated enzymes (Reith and Schuler, 1971). On the other hand, centrilobular cells have higher levels of glycolytic enzymes and the enzymes of fatty acid oxidation (Novikoff, Shin and Drucker, 1960; Novikoff and Essner, 1960; Desmet, 1963).

The administration of a number of hepatocarcinogens including N,N-dimethyl-4-aminoazobenzene (DAB), the carcinogen which is used in this study, has been shown to result in some cell death and a form of regeneration which disorganizes the normal architecture of the liver. This is associated with the appearance of new cell types, for example " oval

* Present address: The Mathilda and Terence Kennedy Institute of Rheumatology, Bute Gardens, London W6 7DW.

cells" which originate in the portal region and later, abnormal parenchymal cells which are distributed more generally (Price *et al.*, 1952; Farber, 1956; Steiner and Carruthers, 1961; Grisham and Hartroft, 1961; Rubin, 1964; Daoust and Molnar, 1964; Molnar and Daoust, 1965). With time, foci of neoplastic cells appear among this altered cell population (Desmet, 1963).

Isolated liver cells have been prepared by a number of methods (Mateyko and Kopac, 1963; Jacob and Bhargava, 1962; Ontko, 1967; Rappaport and Howze, 1966*a,b,c*; Gerschenson and Casanello, 1968; Pertoft, 1969; Castagna and Chauveau, 1963), of which the most successful involve perfusion of the liver with $Ca^{2+}$-free media containing collagenase and hyaluronidase (Howard *et al.*, 1967; Howard and Pesch, 1968; Berry and Friend, 1969; Seglen, 1972, 1973*a,b*; Howard, Lee and Pesch, 1973; Quirstoff, Bondesen and Grunnet, 1973; Ingebretson and Wagle, 1972). The differences in size and composition of cells occurring within the lobule could possibly provide the basis of a fractionation of liver cells and 2 laboratories have already reported fractionations of isolated cells based on this supposition (Castagna and Chauveau, 1969; Castagna *et al.*, 1969; Walter *et al.*, 1973).

In the present study we have separated livers from normal and carcinogen treated animals into readily dissociated cells and tissue residue. The readily dissociated cells have been separated into 3 parenchymal cell fractions by centrifugation. This paper describes some of the characteristics of the different cell populations which have been obtained.

### MATERIALS AND METHODS

*Animals and diets.*—Adult male Wistar rats inbred in this Institute were used. Their weight at the start of feeding experiments was approximately 200 g. Animals referred to as normal were allowed access *ad libitum* to a standard diet of Rowett 86 pellets (Thompson, 1936) and water. Animals for carcinogen treatment were fed a synthetic 10% protein diet containing 0·06% DAB (Miller *et al.*, 1948) for periods from 2 to 10 weeks. The DAB was purchased from British Drug Houses, Poole, Dorset.

*Preparation of isolated cells.*—The method of dissociation of liver cells was based on that of Berry and Friend (1969). Animals were anaesthetized with Nembutal (pentobarbitone sodium) administered intraperitoneally and given 0·1 ml heparin (500 i.u.) intravenously. The portal and hepatic veins of the liver were cannulated and the liver perfused at the rate of 16–18 ml/min with oxygenated $Ca^{2+}$-free Hanks' solution (Hanks and Wallace, 1949) containing collagenase and hyaluronidase. Collagenase was obtained either from the Worthington Biochemical Corporation, N.J., U.S.A. or the Boehringer Corporation, U.K. Hyaluronidase type I was purchased from Sigma, London, U.K. The perfusate was recirculated for 20 min, after which time the liver was chopped into pieces and incubated with the perfusion medium containing the enzymes for 15 min in order to produce maximal dissociation of cells. This resulting suspension of dissociated and undissociated tissue was then filtered through nylon mesh of pore size 60 $\mu$m supplied by Henry Simon Ltd, Cheadle Heath, Stockport, U.K. During filtration slight pressure was administered to the mesh from the outside with a round-ended glass rod in order to induce the greatest possible number of dissociated cells to pass through the mesh.

The filtrate containing the dissociated cells was cooled to 4°C and all subsequent steps were performed at this temperature.

Firstly, the cell suspension was centrifuged at 50 *g* for 3 min. The resulting pellet was washed twice by resuspension in oxygenated $Ca^{2+}$-free Hanks' solution followed by resedimentation. The final washed pellet was resuspended in 35 ml $Ca^{2+}$-free Hanks' solution. This fraction consisted mainly of whole parenchymal cells and was designated fraction P. All supernatants obtained in the preparation of the P fraction were combined and centrifuged at 50 *g* for 5 min. The resultant small pellet was washed in $Ca^{2+}$-free Hanks' solution and resuspended in a volume of 5–10 ml. This fraction consisted largely of non-parenchymal cells and was referred to as NP.

*Fractionation of the parenchymal cell suspension.*—Seven ml of fraction P were layered over 15 ml of 9·35% (w/v) Ficoll (density, 1·033 g/ml, 4°C) in round-bottomed borosilicate glass tubes (length 9·0 cm, diameter 2·4 cm) and centrifuged at 50 *g* for 3 min. Ficoll was obtained from Pharmacia, U.K. The Ficoll-free layer at the top of the tube, together with the interface, was removed and the remaining 9·35% (w/v) Ficoll medium was divided into equal upper and lower fractions. Both fractions were diluted with $Ca^{2+}$-free Hanks' medium and pelleted by centrifugation at 50 *g* for 3 min. The fraction of cells which sedimented faster in the 9·35% (w/v) Ficoll solution was washed twice in $Ca^{2+}$-free Hanks' solution to remove traces of Ficoll and set aside in suspension in $Ca^{2+}$-free Hanks' solution. These cells were referred to as fast sedimenting (F) cells.

The fraction of cells which sedimented slower in 9·35% (w/v) Ficoll solution was resuspended in approximately 5 ml $Ca^{2+}$-free Hanks' solution and relayered over 15 ml 7·5% (w/v) Ficoll solution (density 1·026 g/ml, 4°C) and again centrifuged at 50 *g* for 3 min. The Ficoll-free layer at the top of the tube, together with the interface, was removed and the remaining 7·5% (w/v) Ficoll medium was divided into equal upper and lower fractions. Both fractions were then sedimented, washed twice in $Ca^{2+}$-free Hanks' solution and resuspended in $Ca^{2+}$-free Hanks' solution. The cells which sedimented slower in 7·5% (w/v) Ficoll solution were referred to as slow sedimenting cells (S) while those which sedimented faster were referred to as intermediate sedimenting cells (I). NP cells were not obtained in sufficient yield for similar fractionation.

*Microscopy.*—Cell counts were performed in a Neubauer double cell counting chamber. Cell and nuclear diameters were measured directly from photomicrographs. Lipid vacuoles were detected by staining fresh smears with oil red O. Tissue residues were fixed in formol–calcium (Baker, 1944) and examined histologically. Isolated cells were fixed either before or after pelleting with phosphate buffered $OsO_4$ at pH 7·3 (Millonig, 1961) embedded in Epon (Luft, 1961) and screened with the electron microscope by Dr R. M. Hicks, Department of Pathology, Middlesex Hospital Medical School, London W1P 7PN.

*Chemical estimations.*—Protein, DNA and RNA were determined by the method of Lowry *et al.* (1951), the diphenylamine method (Burton, 1956) and the orcinol method (Mejbaum, 1939) respectively. Standards were bovine serum albumin (B.D.H., Poole, Dorset), calf thymus DNA type I and bovine testicular RNA type I, both from Sigma, London.

*Acid phosphatase activity.*—$\beta$-glycerophosphatase activity was assayed on 0·25 ml samples of cell suspensions for 1 h using 0·5 mol/l $\beta$-glycerophosphate in 1 mol/l acetate buffer, pH 5·0 as substrate (Bertaght and De Duve, 1952). Inorganic phosphate released into the supernatant was assayed by the method of Fiske and Subbarow (1925).

### RESULTS

### *Dissociation of liver cells*

During perfusion with the enzyme solution the structure of the liver began to break down, with the result that after about 10 min in the case of the normal animal, perfusate began to pass through the surface of the liver. In DAB treated liver this effect was variable and often occurred as soon as 5 min after the commencement of perfusion.

The weight of residue remaining in the nylon mesh indicated that both normal and DAB treated liver were approximately 50% dissociated by the present method.

### *Yield of cells*

The total yields of P and NP cells are shown in Table I. The yield of P cells from DAB treated liver was very variable compared with that from normal liver but there was no statistically significant difference between the two. The yield of NP cells was much less than that of P cells in both cases. Again the yield of NP cells from DAB treated liver was more variable than that from normal cells, but in this case the yield from DAB treated liver compared with normal liver was significantly greater statistically ($P < 0{\cdot}001$).

### *Microscopy*

Light microscopy showed that P cells from normal liver consisted of single, large and well-rounded parenchymal cells of

TABLE I.—*Total Yield of Parenchymal (P) and Non-parenchymal Cells (NP) from Normal and DAB treated Liver*

| | *P* | NP |
|---|---|---|
| Normal | $206 \cdot 6 \pm 41 \cdot 7 \times 10^6$ (6) | $15 \cdot 4 \pm 5 \cdot 0 \times 10^6$ (6) |
| DAB* | $221 \cdot 2 \pm 86 \cdot 0 \times 10^6$ (19) | $40 \cdot 0 \pm 32 \cdot 6 \times 10^6$ (19) |

Values are expressed as means ± s.d. with the number of experiments in parentheses.
* 19 animals were fed DAB diet for periods from 12 to 71 days.

mean diameter approximately 30 $\mu$m. Occasionally small aggregates occurred. P cells from DAB treated liver appeared smaller, with a mean diameter of 25 $\mu$m and many were vacuolated.

NP cells from normal and DAB treated liver were smaller than their corresponding P cells. The mean diameter from normal liver was 24 $\mu$m and from DAB treated liver 18 $\mu$m. As expected, electron microscopy showed that this was a very mixed fraction containing Kupffer cells, plasma cells, bile duct cells, lymphocytes and erythrocytes as well as cell debris.

Tissue residue from normal cells was found to contain large masses of poorly dissociated tissue, clumped dissociated cells, trapped single cells and cell fragments. Many cells were vacuolated and more binucleate cells were observed in the tissue residue than in the cell suspension. The architecture of the poorly dissociated tissue corresponded with that of the periportal region of the intact liver: intact bile ductules and parts of the portal vascular branches with partially dissociated cords of cells attached were identified.

Tissue residue from DAB treated liver appeared less dissociated. Even small fragments contained cords of undissociated cells and connective tissue fibres. Oil red 0 staining indicated the presence of numbers of lipid vacuoles which were rarely seen in normal tissue residue. Portal remnants were more apparent in large fragments of tissue residue from DAB treated liver. Abnormal structures were also present in this tissue residue, such as areas of proliferated " oval " cells and, with longer times of feeding, areas of cholangiofibrosis and neoplastic nodules. Electron microscopic examinations of isolated cells indicated that the fewer manipulations the cells had undergone during their fractionation the better was their apparent state of preservation. Therefore F cells appeared better preserved than S cells. Isolated cells from DAB treated liver showed changes associated with the action of the carcinogen, namely, many free ribosomes and a proliferation of smooth endoplasmic reticulum.

*Quantitative data for cells from normal liver having fast intermediate and slow rates of sedimentation*

The numbers of normal cells recovered in the fractions, F, I and S were in the proportions 5 : 4 : 1 respectively. Data collected for the F, I and S fractions show trends some of which are statistically significant. The trend is best seen by looking at the extremes, namely the F and S fractions. Cell diameter varies in the manner F > S. In all other measurements, namely nucleocytoplasmic ratio, protein, DNA, RNA and acid phosphatase contents per $10^6$ cells there is a converse trend, *i.e.* S > F (see Tables II and III).

Differences between S and F fractions are statistically significant in the cases DNA content ($P < 0{\cdot}005$) and acid phosphatase activity ($P < 0{\cdot}05$).

*Cells from DAB treated liver*

Cells were fractionated from 10 animals receiving diet for 23, 27, 31, 38, 45, 50, 52, 58, 60 and 64 days respectively. In most of the parameters measured no time dependent trend was observed and therefore results were treated statistically *en bloc*.

Numbers of cells in the fractions F, I

TABLE II.—*Relative Yields, Cell Diameters and Nucleocytoplasmic Ratios of Fast (F), Intermediate (I) and Slow (S) Sedimenting Cells from Normal and DAB Treated Livers*

| | Normal | DAB treated | P |
|---|---|---|---|
| *% Yield* | | | |
| F | 49·46±21·65 (5) | 38·4±17·6 (10) | N.S. |
| I | 40·84±17·39 (5) | 48·3±19·0 (10) | N.S. |
| S | 10·48± 4·53 (5) | 16·7±4·83 (10) | <0·05 |
| *Cell diameter* | | | |
| F | 28·5±5·4 μm | 25·6±4·9 μm | N.S. |
| I | 27·0±4·1 μm | 24·5±4·3 μm | N.S. |
| S | 25·4±3·2 μm | 24·1±4·0 μm | N.S. |
| *Nucleocytoplasmic ratio* | | | |
| F | 12·90± 3·44% | 18·54±6·80% | <0·001 |
| I | 15·58± 9·16% | 21·69±8·42% | <0·05 |
| S | 24·69±10·75% | 23·49±8·34% | N.S. |

Cell and nuclear diameters were measured directly from light micrographs of liver cell suspensions from 5 normal animals and 18 DAB treated animals (12–71 days). Nucleocytoplasmic ratio was expressed as the percentage of the total cell area occupied by the nuclear area. Values are expressed as means ± s.d. of 5 fields per experiment.

TABLE III.—*Biochemical Results from Fast (F), Intermediate (I) and Slow (S) Sedimenting Cells from Normal and DAB-Treated Livers*

| | Normal | DAB treated |
|---|---|---|
| *mg Protein/$10^6$ cells* | | |
| F | 1·61±0·27 (5) | 1·56±0·56 (10) |
| I | 1·61±0·24 (5) | 1·28±0·58 (10) |
| S | 1·73±0·12 (5) | 0·91±0·40 (10) |
| *μg DNA/$10^6$* cells | | |
| F | 17·82±3·30 (5) | 25·05± 8·28 (10) |
| I | 19·74±3·94 (5) | 24·55± 9·25 (10) |
| S | 30·65±5·50 (5) | 29·06±17·13 (10) |
| *μg RNA/$10^6$ cells* | | |
| F | 122·75± 3·96 (5) | 92·9±39·81 (10) |
| I | 127·97±13·72 (5) | 79·2±28·93 (10) |
| S | 131·20±18·10 (5) | 57·6±19·53 (10) |
| *μg P released/60/$10^6$ cells* (acid phosphatase activity) | | |
| F | 56·66±25·44 (5) | 36·3 ±19·27 (10) |
| I | 60·91±21·19 (5) | 40·6 ±18· 5 (10) |
| S | 97·80±24·22 (5) | 29·75±22·44 (10) |

Figures are expressed as mean ± s.d. The numbers of experiments are in parentheses.

and S occurred in the proportions 4 : 5 : 1·7 respectively.

Cell diameter, nucleocytoplasmic ratio, acid phosphatase activity and DNA content differed little between the fractions. However, protein and RNA contents varied in the manner F > S and the differences were statistically significant, $P$ being $< 0·005$ for protein content and $< 0·05$ for RNA content (see Tables II and III).

*Comparison of normal cells and cells from DAB treated liver*

Statistically significant differences were found in some comparisons between the same fractions of normal cells and cells from DAB treated liver: For example, cells from DAB treated liver sedimented more slowly. The S fraction from DAB treated animals had significantly higher numbers of cells than that from normal animals ($P < 0·05$) and the much larger numbers of cells in the NP fraction from DAB treated liver ($P < 0{\cdot}05$) was attributed to an increase in the small parenchymal cells in this fraction. Except in the case of S cells, DAB treatment gave rise to cells with a higher nucleocytoplasmic ratio (for F cells $P < 0{\cdot}001$ and I cells $P < 0{\cdot}05$) while the protein and RNA contents and acid phosphatase activity were lower in all fractions, but particularly so in the S fraction where in statistical analysis $P < 0{\cdot}001$ in all cases (see Tables II and III).

It is stated above that no time dependent trend was observed in most of the parameters measured. One possible exception, however, was the DNA content of slow cells which was lower than controls at most time intervals but rose to 200% and 170% of the mean control value at 38 and 45 days respectively. This is reflected in the high standard deviation for this group of results shown in Table III.

### DISCUSSION

*Fractionation of normal liver*

The technique used to produce the liver suspension itself brings about a fractionation. Only half the liver is dissociated and when the residue is examined histologically it is composed mainly of periportal areas. From this it is concluded that the dissociated cells are largely of centrilobular and mediolobular origin.

The NP fraction contains blood cells, small parenchymal cells of periportal origin and some bile duct cells. It contains very few Kupffer cells, despite the fact that these cells are numerous (36% cell number in the liver), have no intercellular junctions and are relatively resistant to proteolytic enzymes (Mills and Zucker-Franklin, 1969). The work of Van Berkel and Seglen suggests that a higher centrifugal force than used in the present work is required to sediment them (Seglen, 1973*b*; Van Berkel, 1974).

The F, I and S parenchymal cells appear to indicate a fractionation of cells according to their origin in the lobule. The cells have been separated according to size and nucleocytoplasmic ratio. S parenchymal cells are small, have a high nucleocytoplasmic ratio, a high DNA content and a high acid phosphatase level. All these properties are characteristic of periportal cells, as described by histologists and histochemists (Novikoff *et al.*, 1960; Novikoff and Essner, 1960; Desmet, 1963). Their yield is also low, which is to be expected from a method giving poor dissociation of periportal cells.

The F and I cells presumably represent a range of cells from the centrilobular and mediolobular regions. A method has been described by Seglen (1973*b*) which gives a tissue residue of only 10–15% of the total liver weight. A similar fractionation carried out on such dissociated livers would be expected to give higher yields of fraction S.

*Fractionation of DAB treated liver*

The fractionation of livers of animals which had received DAB was different from that of normal liver at every point. Although 50% of the liver still remained undissociated, the residue from DAB treated liver showed even more cells associated as cords of liver cells. Also in this residue abnormal structures of a ductular nature were sometimes found. At later stages of feeding when nodules were visible in the liver these failed to dissociate and were also found in the tissue residue.

The P cells from DAB treated liver were somewhat smaller than normal P cells and many of them were vacuolated and contained lipid droplets, which is a toxic effect occurring during DAB administration (Porter and Bruni, 1959; Ketterer, Holt and Ross-Mansell, 1967; Bruni, 1960; Svoboda and Higginson, 1968). When submitted to the same F, I and S subfractionation as normal P cells, it is clear that they form a very different population of cells. Instead of this fractionation being determined by size and nucleocytoplasmic ratio, differences in these characteristics in the F, I and S fractions are small. The differences between these which are most marked are in RNA and protein content. After DAB treatment acid phosphatase levels in the whole liver have been shown to fall (Desmet, 1963) and the F, I and S fractions all have similar low levels of this enzyme, which is apparently no longer an index of the periportal origin of a cell.

These differences are statistically significant and refer to the results from all DAB treated animals grouped together. However, a possible peak in DNA content was observed at 38 and 45 days. This period of time requires further study in order to determine whether changes occur at this time which are statistically significant. It could represent a period of DNA synthesis preceding cell proliferation (see Price *et al.*, 1952).

Histologists have noted the presence of nests of hyperbasophilic cells and cells with polyploid nuclei in carcinogen treated livers and have suggested that these are foci for future neoplasia (Desmet, 1963; Daoust and Molnar, 1964; Molnar and Daoust, 1965). However, since these cells probably account for a small proportion of the total cells, they are not likely to be detectable in the fractions obtained in this work.

The cells fractionated resemble small parenchymal cells and show signs of toxic effects, in that they are vacuolated and have lipid inclusions. Under the electron microscope, the presence of free ribosomes is seen and in addition there is massive proliferation of smooth endoplasmic reticulum. The few biochemical characteristics we have measured show how different these cells are from normal cells. It is clear that they are cells responding to the carcinogen but it cannot be said from the simple data available whether or not any of these cell fractions show changes characteristic of stages leading to preneoplasia. More sophisticated experiments which might reveal such changes are feasible. For example, it would be of interest, using tracer labelled compounds, to study carcinogen binding, nuclease activity, DNA and RNA turnover and rate of protein synthesis in the 3 cell fractions and in the tissue residue.

In conclusion, this preliminary survey shows that the method of liver cell dissociation utilizing perfusion with collagenase and hyaluronidase as used in the present work tends to leave periportal tissue undissociated. Of the cells which do dissociate, a potentially useful fractionation of cells can be obtained from both normal and DAB treated liver using rate sedimentation in Ficoll solutions at low *g* for short periods of time.

This work was made possible by a generous grant from the Cancer Research Campaign. We would like to thank Miss Lucia Christodoulides for technical assistance and Dr T. Powell for reading the manuscript and useful discussions.

REFERENCES

Baker, J. R. (1944) The Structure and Chemical Composition of Golgi Element. *Q. J. microscop. Sci.*, **85**, 1.

Berry, M. N. & Friend, D. S. (1969) High Yield Preparation of Isolated Rat Liver Parenchymal Cells. A Biochemical and Fine Structural Study. *J. cell Biol.*, **43**, 506.

Bertaght, J. & De Duve, D. (1952) Tissue Fractionation Studies. 1. The Existence of a Mitochondria-linked Enzymically Inactive Form of Acid Phosphatase in Rat Liver Tissue. *Biochem. J.*, **50**, 174.

Bruni, C. (1960) Hyaline Degeneration of Rat Liver Cells Studied with the Electron Microscope. *Lab. Invest.*, **9**, 209.

Burton, K. (1956) A Study of the Conditions and Mechanism of the Diphenylamine Reaction for the Colorimetric Estimation of Deoxyribonucleic Acid. *Biochem. J.*, **62**, 315.

Castagna, M. & Chauveau, J. (1963) Dispersion du tissu hépatique a l'état de cellules isolées. *C.R. Acad. Sci.*, **257**, 969.

Castagna, M. & Chauveau, J. (1969) Séparation des hépatocytes isolés de rat en fractions cellulaires métaboliquement distinctes. *Expl cell Res.*, **57**, 211.

Castagna, M., Chauveau, J., Loireau, M. P. & De Recondo, A. M. (1969) Isolement sur gradient de densité d'une fraction d'hépatocytes intervenant de façon prépondérante dans l'hypertrophie compensatrice chez le rat. *Expl cell Res.*, **57**, 365.

Daoust, R. & Molnar, F. (1964) Cellular Populations and Mitotic Activity in Rat Liver Parenchyma during Azo-dye Carcinogenesis. *Cancer Res.*, **24**, 1898.

DESMET, V. (1963) *Experimentale levercarcinogenese: Histochemische Studie.* Brussel, Arcia Uitgaven N.V. Brussels: Presses Académiques Européennes S.C.

ELIAS, H. (1963) *The Liver—Morphology, Biochemistry and Physiology.* Ed. Ch. Rouiller. London: Academic Press. Vol. 1. p. 45.

FARBER, E. (1956) Similarities in the Sequence of Early Histological Changes Induced in the Liver of the Rat by Ethionine, 2-acetylaminofluorene and 3′-methyl-4-dimethylamino-azobenzene. *Cancer Res.*, **16**, 142.

FISKE, C. H. & SUBBAROW, Y. (1925) The Colorimetric Determination of Phosphorus. *J. biol. Chem.*, **66**, 375.

GERSCHENSON, L. E. & CASANELLO, D. E. (1968) Metabolism of Rat Liver Cells Cultured in Suspension: Insulin and Glucagon Effects on Glycogen Level. *Biochem. biophys. Res. Commun.*, **33**, 584.

GREENGARD, O., FEDERMAN, M. & KNOX, W. E. (1972) Cytomorphometry of Developing Rat Liver and its Application to Enzymic Differentiation. *J. cell Biol.*, **52**, 261.

GRISHAM, J. W. & HARTROFT, W. S. (1961) Morphologic Identification by Electron Microscopy of " Oval " Cells in Experimental Hepatic Degeneration. *Lab. Invest.*, **10**, 317.

HANKS, J. H. & WALLACE, R. E. (1949) Relation of Oxygen and Temperature in Preservation of Tissues by Refrigeration. *Proc. Soc. exp. Biol. Med.*, **71**, 196.

HOWARD, R. B. & PESCH, L. A. (1968) Respiratory Activity of Intact Isolated Parenchymal Cells from Rat Liver. *J. biol. Chem.*, **243**, 3105.

HOWARD, R. B., CHRISTENSEN, A. K., GIBBS, F. A. & PESCH, L. A. (1967) The Enzymatic Preparation of Isolated Intact Parenchymal Cells from Rat Liver. *J. cell Biol.*, **35**, 675.

HOWARD, R. B., LEE, J. C. & PESCH, L. A. (1973) The Fine Structure, Potassium Content and Respiratory Activity of Isolated Rat Liver Parenchymal Cells Prepared by Improved Enzymatic Techniques. *J. cell Biol.*, **57**, 642.

INGEBRETSON, W. R. & WAGLE, S. R. (1972) A Rapid Method for the Isolation of Large Quantities of Rat Liver Parenchymal Cells with High Anabolic Rates. *Biochem. biophys. Res. Commun.*, **47**, 403.

JACOB, S. T. & BHARGAVA, P. M. (1962) A New Method for the Preparation of Liver Cell Suspensions. *Expl cell Res.*, **27**, 453.

KETTERER, B., HOLT, S. J. & ROSS-MANSELL, P. (1967) The Effect of a Single Intraperitoneal Dose of the Hepatocarcinogen 4-dimethylaminoazobenzene on the Rough Surfaced Endoplasmic Reticulum of the Liver of the Rat. *Biochem. J.*, **103**, 692.

LOWRY, O. H., ROSEBROUGH, N. J., FARR, A. L. & RANDELL, R. J. (1951) Protein Measurement with the Folin Phenol Reagent. *J. biol. Chem.*, **193**, 265.

LUFT, J. H. (1961) Improvements in Epoxy Resin Embedding Methods. *J. biophys. biochem. Cytol.*, **9**, 409.

MATEYKO, G. M. & KOPAC, M. J. (1963) Cytophysical Studies on Living Normal and Neoplastic Cells. *Ann. N.Y. Acad. Sci.*, **105**, 183.

MEJBAUM, W. (1939) Über die bestimmung kleiner pentosemengen insbesondere in derivaten der adenylsäure. *Z. physiol. Chem.*, **258**, 117.

MILLER, E. C., MILLER, J. A., KLINE, B. E. & RUSCH, H. P. (1948) Correlation of Level of Hepatic Riboflavin with Appearance of Liver Tumours in Rats Fed Aminoazo Dyes. *J. exp. Med.*, **88**, 89.

MILLONIG, G. (1961) A Modified Procedure for Lead Staining of Thin Sections. *J. biophys. biochem. Cytol.*, **11**, 736.

MILLS, D. M. & ZUCKER-FRANKLIN, D. (1969) Electron Microscopic Study of Isolated Kupffer Cells. *Am. J. Path.*, **54**, 147.

MOLNAR, F. & DAOUST, R. (1965) Nucleocytoplasmic Ratios in Different Populations of Rat Liver Parenchymal Cells during Azo-dye Carcinogenesis. *Cancer Res.*, **25**, 1213.

NOVIKOFF, A. B. & ESSNER, E. (1960) The Liver Cell. Some New Approaches to its Study. *Am. J. Med.*, **29**, 102.

NOVIKOFF, A. B., SHIN, W. Y. & DRUCKER, J. (1960) Cold Acetone Fixation for Enzyme Localization in Frozen Sections. *J. Histochem. Cytochem.*, **8**, 37.

ONTKO, J. A. (1967) Chylomicron, Free Fatty Acid and Ketone Body Metabolism of Isolated Liver Cells and Liver Homogenates. *Biochim. biophys. Acta*, **137**, 13.

PERTOFT, H. (1969) The Separation of Rat Liver Cells in Colloidal Silica-Polyethylene Glycol Gradients. *Expl cell Res.*, **57**, 338.

PORTER, K. R. & BRUNI, C. (1959) An Electron Microscope Study of the Early Effects of 3′-Me-DAB on Rat Liver Cells. *Cancer Res.*, **19**, 997.

PRICE, J. M., HARMAN, J. W., MILLER, E. C. & MILLER, J. A. (1952) Progressive Microscopic Alterations in the Livers of Rats Fed the Hepatic Carcinogen 3′-methyl-4-dimethylaminoazobenzene and 4′-fluoro-4-dimethylaminoazobenzene. *Cancer Res.*, **12**, 192.

QUIRSTOFF, B., BONDESEN, S. & GRUNNET, N. (1973) Preparation and Biochemical Characterization of Parenchymal Cells from Rat Liver. *Biochim. biophys. Acta*, **320**, 503.

RAPPAPORT, A. M. & BILBEY, D. L. J. (1960) Segmentation of the Liver at Microscopic Level. *Anat. Rec.*, **136**, 262.

RAPPAPORT, C. & HOWZE, G. B. (1966*a*) Dissociation of Adult Mouse Liver by Sodium Tetraphenylboron, a Potassium Complexing Agent. *Proc. Soc. exp. Biol. Med.*, **121**, 1010.

RAPPAPORT, C. & HOWZE, G. B. (1966*b*) Further Studies on the Dissociation of Adult Mouse Tissue. *Proc. Soc. exp. Biol. Med.*, **121**, 1016.

RAPPAPORT, C. & HOWZE, G. B. (1966*c*) Effect of Temperature on the Dissociation of Adult Mouse Liver with Sodium Tetraphenylboron (TPB). *Proc. Soc. exp. Biol. Med.*, **121**, 1022.

REITH, A. & SCHULER, B. (1971) Demonstration of Cytochrome Oxidase Activity with Diaminobenzidine. A Biochemical and Electron Microscopic Study. *J. ultrastruct. Res.*, **36**, 550.

RUBIN, E. (1964) The Origin and Fate of Proliferated Bile Ductular Cells. *Expl molec. Pathol.*, **3**, 279.

SEGLEN, P. O. (1972) Preparation of Rat Liver Cells. I. Effect of $Ca^{2+}$ on Enzymatic Dispersions of Isolated, Perfused Liver. *Expl cell Res.*, **74**, 450.

SEGLEN, P. O. (1973*a*) Preparation of Rat Liver Cells. II. Effects of Ions and Chelators on Tissue Dispersion. *Expl cell Res.*, **76**, 25.

SEGLEN, P. O. (1973*b*) Preparation of Rat Liver Cells. III. Enzymatic Requirements for Tissue Dispersion. *Expl cell Res.*, **82**, 391.

STEINER, J. W. & CARRUTHERS, J. S. (1961) Studies on the Fine Structure of the Terminal Branches of the Biliary Tree. I. The Morphology of Normal Bile Canaliculi, Bile Preductules (ducts of Hering) and Bile Ductules. *Am. J. Path.*, **38**, 639.

SVOBODA, D. & HIGGINSON, J. (1968) A Comparison of Ultrastructural Changes in Rat Liver due to Chemical Carcinogens. *Cancer Res.*, **28**, 1703.

THOMPSON, W. (1936) Stock Diet for Rats. *J. Hyg. Camb.*, **36**, 24.

VAN BERKEL, TH. J. C. (1974) Difference Spectra, Catalase and Peroxidase Activities of Isolated Parenchymal and Non-parenchymal Cells from Rat Liver. *Biochem. biophys. Res. Commun.*, **61**, 204.

WALTER, H., KROB, E. J., ASCHER, G. S. & SEAMAN, G. V. F. (1973) Partition of Rat Liver Cells in Aqueous Dextran–Polyethylene Glycol Phase Systems. *Expl cell Res.*, **82**, 15.